# A Case of Severe Asthma with Eosinophilic Otitis Media Successfully Treated with Anti-IgE Monoclonal Antibody Omalizumab

**DOI:** 10.1155/2012/340525

**Published:** 2012-11-25

**Authors:** Azusa Okude, Etsuko Tagaya, Mitsuko Kondo, Manabu Nonaka, Jun Tamaoki

**Affiliations:** ^1^First Department of Medicine, School of Medicine, Tokyo Women's Medical University, 8-1 Kawada-Cho, Shinjuku-ku, Tokyo 162-8666, Japan; ^2^Department of Otolaryngology, School of Medicine, Tokyo Women's Medical University, 8-1 Kawada-Cho, Shinjuku-ku, Tokyo 162-8666, Japan

## Abstract

A 51-year-old woman had been receiving medical treatment for asthma since she was 21 years old. However, her asthma was poorly controlled despite treatment involving combination inhalation of high-dose corticosteroid and long-acting *β*_2_-aderenergic agonist (LABA) and regularly taking oral steroids. Hearing loss and otorrhea appeared at the age of 44, and she was given a diagnosis of eosinophilic otitis media (EOM) and received medical treatment. In 2007, symptoms of asthma and otitis media deteriorated. In December 2009, omalizumab therapy was started for refractory asthma. After 2 months of omalizumab treatment, not only asthma, but also hearing loss improved. It is suggested that early initiation of omalizumab therapy may inhibit the progression of progressive EOM.

## 1. Introduction

The anti-immunoglobulin E (IgE) monoclonal antibody omalizumab was approved in 2005, by the European Medicines Agency for the treatment of severe asthma [1]. In Japan, omalizumab has been used since 2009, for treating very severe persistent asthma [2]. Omalizumab is expected to exhibit a therapeutic effect on medical conditions associated with eosinophilic inflammation. However, since the approved indication of omalizumab is intractable asthma, very little has been reported about the efficiency of omalizumab on EOM. We describe a case of severe asthma and concurrent intractable EOM in which omalizumab therapy improved the symptoms of both asthma and EOM.

## 2. Case Report

A 51-year-old woman presented with chief complaints of wheezing and hearing loss. She was a nonsmoker with a history of allergic rhinitis. At 21 years of age, bronchial asthma was diagnosed, and she has been receiving medical treatment ever since. However, because the asthma was poorly controlled, she had been regularly taking inhaled corticosteroid (ICS), LABA, antileukotriene agents, and oral steroids (prednisolone (PSL), 10–15 mg/day).

When she was 44 years old, she started to experience symptoms of hearing loss and otorrhea. She was treated with ear drops containing steroids; however, her hearing loss gradually worsened. She was diagnosed with EOM because she had recurrent asthma attacks and otitis media with highly viscous middle ear effusion. PSL dose was increased to 40 mg/day. Subsequently, the steroid dosage was reduced; however, her asthma was poorly controlled, and symptoms of otorrhea and hearing worsened. Omalizumab (Novartis Pharma K.K., Tokyo, Japan) treatment for severe asthma was started from December 12, 2009. Physical findings: Body mass index (BMI) was 21.3 kg/m^2^. Rhonchi were heard from the right lung field. Hearing loss was profound in both ears.

## 3. Clinical Laboratory Results (Table 1)

The patient was receiving 10 mg/day PSL; no increase was observed in the peripheral blood eosinophil count. The total IgE level was 97.4 IU/mL. The results of the radioallergosorbent test (RAST) were positive for Japanese cedar, *Dermatophagoides pteronyssinus*, *Dermatophagoides farinae*, and house dust. The patient tested negative for antineutrophil cytoplasmic antibodies (ANCAs). Chest X-ray showed no abnormal findings. The patient had no symptoms indicative of allergic bronchopulmonary aspergillosis (ABPA) or Churg-Strauss syndrome.

## 4. Clinical Course

**Figure 1:**
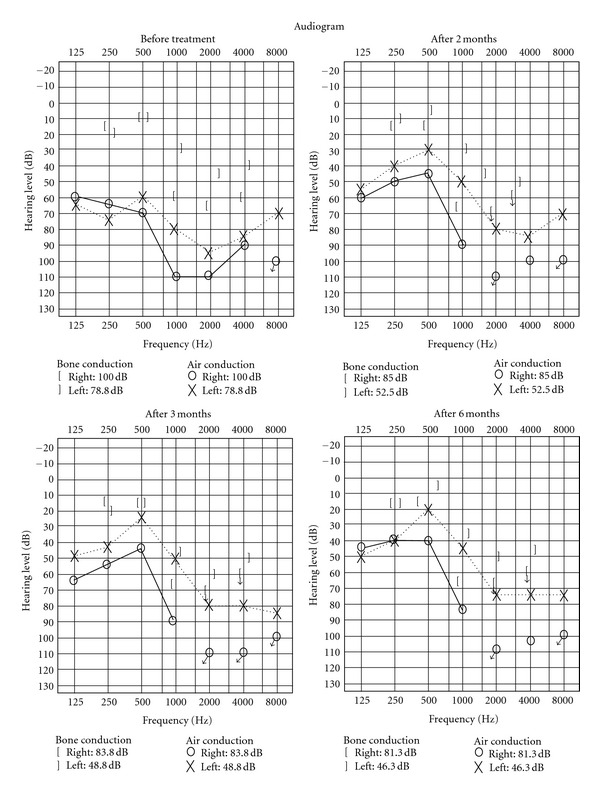
The changes of the audiogram before and after omalizumab treatment. Before omalizumab treatment, the patient had developed impaired hearing both by air and bone conduction; however, after the second omalizumab administration, air conduction hearing improved in the low-frequency range in both ears.

Total IgE level slightly increased due to omalizumab therapy. Regarding lung function, an increase in the mean peak expiratory flow (PEF), along with improvement in the FEV_1_/FVC, was observed at 2 months and 4 months after initiating omalizumab therapy. The use of short-acting *β*_2_ stimulant (SABA) was significantly decreased (Table 2). Because improvement was observed at 4 months after the start of omalizumab therapy, PSL dose was reduced. However, after 1 year, exacerbation of asthma symptoms with concurrent infection was observed, with simultaneous PEF decrease. The audiogram indicated that, before omalizumab treatment, the patient had developed impaired hearing both by air and bone conduction; however, after the second omalizumab administration, air conduction hearing improved in the low-frequency range in both ears. Improved hearing has been maintained (Figure 1). Amelioration of both asthma symptoms and hearing has contributed to a better quality of life (QOL) of the patient. 

## 5. Discussion

Omalizumab is an anti-IgE monoclonal antibody, which binds to free IgE in the blood and inhibits the binding between Fc*ε*RI and IgE on the mast cell membrane. The inhibition of the binding between Fc*ε*RI and IgE prevents degranulation of inflammatory mediators such as histamine and interleukins from the mast cells, thereby inhibiting the allergic response [2]. Although omalizumab is indicated for the treatment of severe asthma, omalizumab is expected to have a therapeutic effect on conditions associated with eosinophilic inflammation.

EOM, first described by Matsutani et al. in 1995 [3], is an intractable otitis media characterized by heavy eosinophilic infiltration and high viscous mucoid effusion, and it is found in patients with adult-onset asthma. The essential diagnostic criterion includes otitis media with effusion in which the middle ear fluid contains eosinophils. A definitive diagnosis is established when the aforementioned condition is observed in conjunction with bronchial asthma, nasal polyps, history of dural incision, or resistance to antimicrobial agents (Table 3) [4]. Although the cause of EOM is not clear, considering that the middle ear fluid contains a high concentration of eosinophil cationic protein (ECP) derived from eosinophils [5] and the report by Nonaka et al. [6] on the high concentration of interleukin-5 (IL-5), eosinophilic inflammation is thought to be induced in the middle ear cavity by some stimuli through the Eustachian tube. The clinical features of EOM are that women aged 40–69 years are most susceptible, and EOM affects both ears in about 80% of causes. EOM occurs in conjunction with bronchial asthma, nasal polyps, or sinusitis. In particular, EOM is found in 90% of patients with bronchial asthma [7]. EOM is often intractable, and can lead to total deafness.

In EOM cases, the biopsy of the middle ear mucous membrane shows eosinophilic infiltration, goblet cell metaplasia, granulation, and fibril formation in the epithelial and subepithelial parts. The clinical features of EOM, that is, high concentration of eosinophils in the middle ear fluid, eosinophilic infiltration in the middle ear mucous membrane, goblet cells metaplasia, and fibril formation in the advanced disease stage, are similar to those of asthma in which eosinophilic inflammation occurs in the lower respiratory tract.

In the present case, although omalizumab was used to treat severe asthma, it improved not only the asthma, but also the EOM. During the first time, 150 mg/dose of omalizumab was administered at 4-week intervals, according to the pretreatment IgE concentration. However, weakening of the therapeutic effect was often observed as early as 2 weeks after treatment initiation. Thus, the dose of omalizumab was increased to 300 mg on the concentration basis of IgE measured before steroid therapy. After increasing the doses, the symptoms improved significantly, and the therapeutic effect was maintained.

The IgE concentration often decreased in patients with severe asthma who are measured before omalizumab therapy [8]. In the present case, the pretreatment IgE concentration was 97.4 IU/mL, which was rounded to 100 IU/mL, and the dose of 150 mg was selected first; however, the dose was later increased to 300 mg to obtain a better result. Hence, upward dose adjustment may improve the clinical outcome. 

## 6. Conclusions

Omalizumab improved the asthma symptoms and hearing in a patient with severe persistent asthma with interactive EOM and progressive hearing loss. In patients with severe asthma, treatment of the complication symptoms is indispensable. Early initiation of omalizumab therapy may inhibit the progression of complicated EOM.

## Figures and Tables

**Table 1:** Clinical laboratory results.

(i) Blood cell count			
WBC	9400/*μ*L	IgE (RIST) 97.4 lU/mL	
Neutro	78.0%	(RAST)	
Lymph	11.5%	Japanese cedar	2+
Mono	7.0%	*Dermatophagoides pteronyssinus *	3+
Eos	0.0%	*Dermatophagoides farina *	2+
Baso	0.0%	House dust	3+
RBC	4.64 × 10^6^/*μ*L		
Hb	11.7 g/dL	MPO-ANCA <10 EU	
Ht	37.9%	PR3-ANCA <10 EU	
Plt	34.8 × 10^4^/*μ*L		
(ii) Blood chemistry			
Alb	3.7 g/dL		
AST	16 U/L		
ALT	19 U/L		
BUN	12.5 mg/dL		
Cr	0.57 mg/dL		
CRP	0.04 mg/dL		
FBS	102 mg/dL		

**Table 2:** The clinical course before and after omalizumab treatment.

Omalizumab dose pack (months)	Before treatment	Post 2 months (5~8 W)	Post 4 months (13~16 W)	Post 6 months (21~24 W)	After 1 year
IgE (IU/mL)	97.4	181	159	144	102
PEF (L/sec): morning/evening	210/290	280/330	280/310	220/280	230/320
PSL dose (mg/day)	15	10	10	10/5	15
Rescue frequency (times/28 day)	12	0	0	7	10
Daily life (days/28 days)					
No difficulty	0	5	28	16	4
Slight difficulty	19	23		7	24
Extreme difficulty	9			5	
Deep sleep (days/28 days)	20	28	28	24	28
Cough Mild	15	14	8	7	9
(days/28 days) strong	3	0	1	5	0
none	9	14	19	16	16
No symptom days (days/28 days)	20	28	27	21	25
Lung function: FEV_1_ (L)	1.93		2.34		
% FEV_1_ (% )	63.9		76.4		
PEF (L/sec)	4.89		5.84		

**Table 3:** Diagnostic criteria of eosinophilic otitis media.

(i) Major: otitis media with effusion or chronic otitis media with eosinophil-dominant effusion
(ii) Minor
(1) Highly viscous middle ear effusion
(2) Resistance to conventional treatment for otitis media
(3) Association with bronchial asthma
(4) Association with nasal polyposis

Definitive case: major + two or more minor criteria.

Exclusion criteria: Churg-Strauss Syndrome and hypereosinophilic syndrome.
